# A never ending race for new and improved fluorescent proteins

**DOI:** 10.1186/1741-7007-10-39

**Published:** 2012-05-03

**Authors:** Alexander M Jones, David W Ehrhardt, Wolf B Frommer

**Affiliations:** 1 Carnegie Institution for Science, 260 Panama St, Stanford, CA 94305, USA

## Abstract

Bioluminescent and fluorescent proteins are now used as tools for research in all organisms. There has been massive progress over the past 15 years in creating a palette of fluorescent proteins with a wide spectrum of specific properties. One of the big challenges is to decide which variant may be best for a certain application. A recent article by Mann *et al*. in *BMC Biotechnology *describes a new orange fluorescent protein in plants.

See research article http://www.biomedcentral.com/1472-6750/12/17

## Text

Fluorescence and bioluminescence occur in terrestrial and aquatic organisms, but are especially common in the marine environment, where they serve diverse functions such as detecting or luring prey in dark environments, the attraction of mates, or a means to evade predation [1,2]. Proteins are key to these phenomena and the first autofluorescent proteins were identified in the crystal jelly *Aequorea victoria *[3]. The subsequent impact on biological research of these fluorescent proteins (FPs), with *A*. *victoria *green FP (avGFP) as the founding member, cannot be overstated. The great importance of FPs in biological research was recognized with the Nobel Prize in Chemistry to Osamu Shimomura, Martin Chalfie, and Roger Y Tsien in 2008 [4]. In a study just published in *BMC Biotechnology*, Mann *et al*. [5] compare orange FP variants that were expressed transiently and stably in plants, raising the issue of what questions need to be asked in the selection of fluorophores. Here we summarize the key questions that need to be addressed to find an optimal fluorophore for a given application in plant biology and beyond.

## Fluorescent proteins as tools for exploring live cell processes

FPs are now indispensable tools for studying dynamic processes in living organisms, tissues and cells [3,6]. They have been exploited for diverse purposes, including as reporters of cell-specific gene expression, for visualizing the development and dynamic cellular architecture of complex tissues like brains (for example, Brainbow), and, perhaps most commonly, for determining the subcellular localization and dynamics of proteins. They are also used to observe and quantify trafficking of proteins to the nucleus, the reshaping of the cytoskeleton, delivery of proteins to the plasma membrane, action of molecular motors, motility of membrane proteins, assembly and disassembly of protein complexes and the turnover of proteins. Today, a full palette of color variants is available [3,6,7], which has been key to the development of biosensors (both single fluorophore and Förster Resonance Energy Transfer (FRET) sensors) that, for the first time, have enabled monitoring of small molecules and metabolites with subcellular resolution, as, for example, glutamate release from neurons [8]. Similarly, they have been used to create sensors for protein activity (reviewed in [8]). More sophisticated versions of these FPs have even laid the path to breaking Ernst Abbe's Law for optical resolution in PALM super-resolution microscopy [9].

Still, the race is on to develop new and improved versions with properties that will help the researcher explore deeper questions. Parameters targeted for improvement include brightness (increased extinction coefficients and quantum yields), novel or narrowed spectral ranges for absorption and emission, improved photostability, increased or decreased oligomerization tendency, faster fluorophore maturation time and reduced sensitivity to environmental fluctuations such as pH changes (Table 1). In addition to engineering efforts designed to modulate the emission spectra, FP mutagenesis has also targeted the separation distance between absorption and emission maxima (Stokes shift) to generate better probes for FRET, fluorescence cross-correlation spectroscopy (FCCS) and multicolor imaging.

## Fluorescent proteins in plants

The use of FPs has revolutionized plant biology just as it has other fields, but plant research offers several specific challenges to effective use of FP-based tools. For example, chlorophyll and stress-induced phenolics often result in significant autofluorescence in the same spectrum as the emission of FPs, which must therefore be spectrally avoided or overcome with brighter signals. Furthermore, rapid work in plant cell culture or transient expression systems is more limited compared to mammalian cell biology (for example, due to massive overexpression when using Agrobacterium-based transient expression in tobacco leaves and lack of plant cell type-specific cell cultures), and analysis of stably transformed individuals is, though time consuming, generally preferred. The work of Mann *et al*. [5] in characterizing fluorescence of stable transgenic plants expressing less utilized bright orange fluorescent proteins (OFPs) helps to overcome some of the challenges of autofluorescence and lowers the barriers to adoption of OFPs. Perhaps we are nearing a point in which plant biologists will rapidly take better advantage of more FP tools as their utility is increasingly demonstrated. Indeed, besides the underutilization of the extensive FP color palette, many advanced FP technologies have yet to be widely adopted in plant research, including a large and growing set of FRET biosensors for quantifying ions and metabolites in live cells with subcellular resolution [8] and photoswitchable FPs for time-sensitive protein dynamics [10]. The availability of multiple colors can be exploited to design orthologous FRET pairs for simultaneous detection of multiple FRET biosensors in live cells [11].

The FPs characterized by Mann and colleagues are among the brightest developed to date. Brighter FPs allow use of lower biosensor expression levels, are critical for detection of low abundance analytes - particularly where there is background from autoflourescence that must be overcome - and also to reduce side effects potentially caused by buffering of ions or metabolites by the biosensor itself. This point will be particularly salient as sensors are developed for very rare signaling molecules such as hormones. Bright FPs also improve detection of low abundance targets above autofluorescent background and are very important for analysis of single molecules and structures labeled with few tagged proteins, such as cellulose synthase and microtubule nucleation complexes [12,13]. Photostability is also very important for studies that push the boundaries of low light imaging, since photobleaching is especially detrimental when only one or a handful of fluorescently tagged proteins are being imaged. Thus, there is a need for FPs with good balances of brightness and photostability, like enhanced (E) GFP. A bright orange protein with EGFP stability or better would be a very valuable tool, especially for use in plants, and should be a priority for FP improvement. Beyond advanced tools, very bright FPs also allow for easy detection with lower quality imaging set-ups and can expand the utility of fluorescence tools into the classroom and the field.

## Which fluorescent protein is best for your experiment?

Mann *et al*. [5] focus on reporters for gene expression levels, in which avoidance of autofluorescence and maximum attainable brightness at the whole cell level are of greatest importance. To evaluate brightness for protein tagging studies, however, output should be evaluated on a per fluorophore basis (that is, fluorophore density or numbers should be measured or estimated). Other applications may favor proteins with other properties [14]. As discussed above, when tracking the dynamic behavior of single proteins or complexes labeled with few fluorophores, photostability may be as important as brightness. For analysis of dynamic gene expression, FPs with rapid folding and increased turnover are important. Studies of proteins in non-cytosolic compartments, like the apoplasm (cell wall space), may require fluorophores that have reduced sensitivity to acidic pH. On the other hand, such sensitivity can be used to design sensors for compartmentalization or membrane protein topology [15,16]. To best evaluate FPs for plant applications, measurements should be performed in the biological context in which the fluorophore will be used. A very useful and missing tool set for such quantitative evaluations of live cell fluorophore performance would be *in vivo *calibration standards for brightness. Such standards have been made for FRET efficiency, but have not yet been applied in plant cells.

**Table 1:** Focus areas for fluorescent protein (FP) optimization

FP property	Examples for relevance	Reference
Reduce or increase sensitivity to environmental changes (for example, pH, ionic conditions)	Reduce sensitivity to gain specificity of response as in metabolite sensors; or increase as in high pH sensitivity of Keima tracer for compartmentalization and membrane topology	[a]

Monomer versus oligomerization	Especially important for environments in which diffusion is constrained like membranes	[b-e]

Folding time	Important for time-sensitive expression studies and some cell biology applications like protein trafficking	[f]

Brightness (quantum yield and extinction coefficient)	Generally, brighter FPs are important for getting signal above high background or when imaging biomolecules or processes that have low abundance (for example, when measuring expression from weak promoters)	[g-i]

Absorption and emission spectra (color change, stokes shift, narrowing of spectra)	Important for avoiding autofluorescence, multicolor imaging and/or specialized imaging modalities such as multiphoton and Stimulated emission depletion microscopy (STED)	[a, j, k-p]

Monoexponential decay	Important for Fluorescence-lifetime imaging microscopy (FLIM)	[g, q]

Photoswitching, photoactivation and photoconversion	Important for monitoring dynamics of proteins and protein populations	[r, s]

Photostability	Important for extended, high-resolution and/or single molecule imaging	[e, t, u]

Footnote: references for the Table:

a. Tantama M, Hung YP, Yellen G: **Imaging intracellular pH in live cells with a genetically encoded red fluorescent protein sensor**. *J Am Chem Soc *2011, **133:**10034-10037.

b. Baird GS, Zacharias DA, Tsien RY: **Biochemistry, mutagenesis, and oligomerization of DsRed, a red fluorescent protein from coral**. *Proc Natl Acad Sci U S A *2000, **97:**11984-11989.

c. Campbell RE, Tour O, Palmer AE, Steinbach PA, Baird GS, Zacharias DA, Tsien RY: **A monomeric red fluorescent protein**. *Proc Natl Acad Sci U S A *2002, **99:**7877-7882.

d. Espagne A, Erard M, Madiona K, Derrien V, Jonasson G, Levy B, Pasquier H, Melki R, Merola F: **Cyan fluorescent protein carries a constitutive mutation that prevents its dimerization**. *Biochemistry *2011, **50:**437-439.

e. Shu X, Lev-Ram V, Olson ES, Aguilera TA, Jiang T, Whitney M, Crisp JL, Steinbach P, Deerinck T, Ellisman MH, Ellies LG, Nguyen QT, Tsien RY: **Spiers Memorial Lecture. Breeding and building molecular spies**. *Faraday Discuss *2011, **149:**9; discussion 63-77.

f. Andrews BT, Gosavi S, Finke JM, Onuchic JN, Jennings PA: **The dual-basin landscape in GFP folding**. *Proc Natl Acad Sci U S A *2008, **105:**12283-12288.

g. Subach OM, Cranfill PJ, Davidson MW, Verkhusha VV: **An enhanced monomeric blue fluorescent protein with the high chemical stability of the chromophore**. *PLoS One *2011, **6:**e28674.

h. Hunt ME, Scherrer MP, Ferrari FD, Matz MV: **Very bright green fluorescent proteins from the Pontellid copepod Pontella mimocerami**. *PLoS One *2010, **5:**e11517.

i. Goedhart J, von Stetten D, Noirclerc-Savoye M, Lelimousin M, Joosen L, Hink MA, van Weeren L, Gadella TW Jr, Royant A: **Structure-guided evolution of cyan fluorescent proteins towards a quantum yield of 93%**. *Nat Commun *2012, **3:**751.

j. Olenych SG, Claxton NS, Ottenberg GK, Davidson MW: **The fluorescent protein color palette**. In *Current Protocols in Cell Biology*. 2008/01/30 edition. Edited by Bonifacino JS, Dasso M, Harford JB, Lippincott-Schwartz J, Yamada KM. Hoboken, NJ: John Wiley & Sons, Inc.; 2007: 21.25.21-21.25.34.

k. Day RN, Davidson MW: **Fluorescent proteins for FRET microscopy: Monitoring protein interactions in living cells**. *Bioessays *2012, **34:**341-350.

l. Mathur J: **The illuminated plant cell**. *Trends Plant Sci *2007, **12:**506-513.

m. Shcherbo D, Murphy CS, Ermakova GV, Solovieva EA, Chepurnykh TV, Shcheglov AS, Verkhusha VV, Pletnev VZ, Hazelwood KL, Roche PM, Lukyanov S, Zaraisky AG, Davidson MW, Chudakov DM: **Far-red fluorescent tags for protein imaging in living tissues**. *Biochem J *2009, **418:**567-574.

n. Zhao Y, Araki S, Wu J, Teramoto T, Chang YF, Nakano M, Abdelfattah AS, Fujiwara M, Ishihara T, Nagai T, Campbell RE: **An expanded palette of genetically encoded Ca(2) indicators**. *Science *2011, **333:**1888-1891.

o. Shcherbakova DM, Hink M, Joosen L, Gadella TW, Verkhusha VV: **An orange fluorescent protein with a large Stokes shift for single-excitation multicolor FCCS and FRET imaging**. *J Am Chem Soc *2012 [Epub ahead of print].

p. Filonov GS, Piatkevich KD, Ting LM, Zhang J, Kim K, Verkhusha VV: **Bright and stable near-infrared fluorescent protein for in vivo imaging**. *Nat Biotechnol *2011, **29:**757-761.

q. Ha T, Tinnefeld P: **Photophysics of fluorescent probes for single-molecule biophysics and super-resolution imaging**. *Annu Rev Phys Chem *2012, **63:**595-617.

r. Markwardt ML, Kremers GJ, Kraft CA, Ray K, Cranfill PJ, Wilson KA, Day RN, Wachter RM, Davidson MW, Rizzo MA: **An improved cerulean fluorescent protein with enhanced brightness and reduced reversible photoswitching**. *PLoS One *2011, **6:**e17896.

s. Mathur J, Griffiths S, Barton K, Schattat MH: **Green-to-red photoconvertible mEosFP-aided live imaging in plants**. *Methods Enzymol *2012, **504:**163-181.

t. Bogdanov AM, Bogdanova EA, Chudakov DM, Gorodnicheva TV, Lukyanov S, Lukyanov KA: **Cell culture medium affects GFP photostability: a solution**. *Nat Methods *2009, **6:**859-860.

u. Shaner NC, Lin MZ, McKeown MR, Steinbach PA, Hazelwood KL, Davidson MW, Tsien RY: **Improving the photostability of bright monomeric orange and red fluorescent proteins**. *Nat Methods *2008, **5:**545-551.
